# Unveiling the burden of premenstrual dysphoric disorder: a narrative review to call for gender perspective and intersectional approaches

**DOI:** 10.3389/fpsyt.2024.1458114

**Published:** 2025-01-21

**Authors:** Dannia Islas-Preciado, Luciana Ramos-Lira, Erika Estrada-Camarena

**Affiliations:** ^1^ Laboratorio de Neuropsicofarmacología, Dirección de Investigaciones en Neurociencias, Instituto Nacional de Psiquiatría “Ramón de la Fuente Muñíz”, Mexico City, Mexico; ^2^ Laboratorio de Neuromodulación, Subdirección de Investigaciones Clínicas, Instituto Nacional de Psiquiatría “Ramón de la Fuente Muñíz”, Mexico City, Mexico; ^3^ Dirección de Investigaciones Epidemiológicas y Psicosociales, Instituto Nacional de Psiquiatría “Ramón de la Fuente Muñíz”, Mexico City, Mexico

**Keywords:** premenstrual dysphoric disorder, burden of illness, gender perspectives, intersectionality, call to action

## Abstract

The present narrative review discusses the burden of Premenstrual Dysphoric Disorder (PMDD) and highlights the lack of awareness by analyzing the following key points: -Prevalence and Diagnosis: PMDD affects a significant portion of women during their reproductive years, but diagnosis is often delayed due to limited understanding and awareness. -Mental Health Burden: PMDD increases the risk of suicide attempts and negatively impacts quality of life. There are also economic costs associated with absenteeism and healthcare use. -Cultural and Gender Perspectives: Societal stigma surrounding menstruation and mental health likely contributes to underdiagnosis. -Lack of Sex and Gender Perspective in the Healthcare System: Research bias towards male subjects and historical neglect of women’s health issues contribute to limited knowledge about PMDD. -Non-Intersectional Approaches: Disparities in access to healthcare and the unique experiences of women further complicate PMDD diagnosis and treatment. -Vicious Cycle: The lack of research and awareness creates a vicious cycle where PMDD remains misunderstood and inaccurately treated. Finally, it emphasizes the need for increased awareness, education, and research on PMDD, particularly with a gendered and intersectional optic. The situation in Latin America is presented as a particular concern due to a lack of recent data and potentially higher prevalence due to socioeconomic factors.

## 1 Introduction

During reproductive age, females are more susceptible than males to suffer mood disorders (1, 2), and there are female-specific psychiatric conditions such as Premenstrual Dysphoric disorder (PMDD), the severe form of premenstrual syndrome (PMS) (3). According to the last version of the Diagnostic and Statistical Manual (DSM-5), PMDD is characterized by depressive mood, anxiety, mood liability, and somatic symptoms that usually appear during the late luteal phase of the menstrual cycle and typically disappear during the first week after menses (3). The symptoms are presented cyclically across the menstrual cycle (4), and there are subtypes of premenstrual symptoms (5) that do not necessarily commit to the temporal course stated in the classical manual diagnosis. Additionally, the DSM-5 criteria require the presence of at least five core symptoms in two consecutive cycles; however, this criterion is not always met in clinical practice. A commonly used diagnostic tool for Premenstrual Dysphoric Disorder (PMDD) is the “Daily Record of Severity of Problems” (DRSP), which prospectively screens for PMDD symptoms (6).

The recent systematic review and meta-analysis of Reilly et al. (7) found a pooled prevalence of 3.2% (95% Confidence Intervals (CI): 1.7%–5.9%) in samples with confirmed diagnosis and 7.7% (95% CI: 5.3%–11.0%) in samples that had provisional diagnosis. Provisional diagnoses are the ones in which the studies did not use prospective symptoms according to DSM criteria (3). When restricted to studies fully adhering to DSM diagnostic criteria for a confirmed diagnosis, in community-based samples, the pooled prevalence was 1.6% (95% CI: 1.0%–2.5%).

Following Reilly et al. (7) location matters, as there was a significant effect of the continent from which the sample was taken (p<0.001), with the highest prevalence in African samples, 27. 8% (95% CI: 14.6%–46.3% and lowest in North American samples, 2.8 (95% CI: 1.7 -4.5). There was a significant effect of sample type (p = 0.007), with the highest prevalence in university samples. It should be noted that all samples from Africa used provisional diagnosis so it may be an overestimation of the true prevalence. Notably, there were not any studies in Latin America, which is *per se* informative of the situation about the omission and overlooking of PMDD in this region. There were only three in Brazil that showed a high prevalence of 13.1 (95% CI: 7.6-21.6), that it is higher than in other zones such as North America.

Interestingly, a meta-analysis (8) reported the prevalence of premenstrual syndrome (PMS) across various regions, including Europe, Asia, Africa, and Brazil. The pooled prevalence of PMS was 47.8% (95% CI: 32.2-62.9), with the lowest prevalence observed in France (12%, 95% CI: 11-13) and the highest in Iran (98%, 95% CI: 97-100). This study highlighted significant regional disparities, with high prevalence rates reported in Iran, Turkey, Pakistan, Nigeria, Brazil, and Spain (up to 50%). The contrast between the high prevalence of Spain and the lower rates in France and Switzerland suggests that cultural and social factors may influence the development of PMS and PMDD.

Supporting this idea, a recent study (9) examined the impact of PMS and PMDD on the academic performance of university students in the United Arab Emirates. A substantial proportion of participants reported experiencing PMS (78.9%) and PMDD (16.3%), indicating that these conditions can significantly affect academic activities in 90% of cases.

Worryingly, symptoms appear around age 15 on average, while the diagnosis occurs on average at age 35 according to a previous report (10). To endure twenty years of not having a diagnosis is a significant health burden to the suffering person. For years, women’s mental health has been undervalued and dismissed (11), leading to delays in diagnosis.

There may be significant health benefits for women to receive timely diagnosis, as women who are diagnosed later in life are more likely to attempt suicide (12). Nevertheless, there are limited options for treatment because of the lack of understanding of the etiology of PMDD, “the lack of ability to test for biomarkers for PMDD, and the complex nature of the behavioral and affective symptoms” (12). However, following Chan et al. (12) demonstrated that healthcare providers often minimize patients’ symptoms, a phenomenon known as “medical gaslighting”. This practice involves a recurring dismissal of symptoms and lack of empathy by healthcare providers.

Overlooking Premenstrual mood disorders can negatively impact the quality of life, and it is imperative to discuss the burden of these disorders, which is often exacerbated by the challenges women face within a healthcare system primarily oriented and designed by men (11). Here, we aimed to review and point out key elements that need to be discussed to raise awareness about premenstrual mood disorders. We will discuss how economic, cultural and societal factors can impact the mental health experiences of women, particularly in developing regions.

## 2 The burden of PMDD in mental health: focus on suicidal risk

It is undoubtedly that a psychiatric condition will impact individuals in various spheres and in different ways. These conditions may lead to a wide-ranging spectrum of outcomes. In this sense, the termination of life comprehends the most fatal consequences in outcomes taxonomy in clinical studies (13). Suicide comprehends ideation, planning, non-fatal and fatal attempts (14). Overall, the literature suggests that men die by suicide in more proportion than women. However, rates of suicide attempts are higher in women than in men (15). As Vijayakumar (15) opportunely points out, there is a significant gap in the literature regarding reports that exhaustively evaluate the complex interplay between gender and suicide. Therefore, gender-perspective studies are necessary to elucidate the complex social, -environmental, and biological demands that may underlie suicide attempts in the female population.

Previous reports have found different risks of suicide attempts and ideation in females with PMDD. For instance, a meta-analysis reported that having PMDD duplicates the risk of suicidal ideation and attempts (16). Moreover, suicidal ideation was reported in 40% of women with PMDD (17). Notably, another meta-analysis that included a higher number of studies (18) found that women with PMDD have 7 times higher risk of suicide attempt (OR= 6.97) and nearly 4 times higher risk of suicide ideation (OR= 3.95) respect to no-PMDD subjects. Eisenlohr-Moul et al. (19) observed higher rates of suicidal ideation in 72% of females with PMDD, while planning in 49%, attempt in 34%. Most participants (92.4%) were females from English-speaking countries, with less than 1% belonging to Latin America regions. While the study unequivocally establishes a connection between suicide and PMDD, the unrepresentative number of participants originating from Latin America regions, limits the ability to draw a perspective on how PMDD is affecting menstruating individuals in this region.

## 3 The economic burden of PMDD

The diagnosis and epidemiology of PMDD have been exhaustively reviewed acknowledging that PMDD is a legitimate disorder deserving of research and clinical attention (20). Being PMDD a health condition, it comes with the so-called burden of disease. The burden of disease refers to the financial costs, mortality, morbidity, and quality of life impact that illnesses bring along (21). For instance, PMDD increases the risk of visiting a specialist physician three times or more during 12 months (22) implying economic costs and resources to receive medical care. Women with PMDD have increased rates of absenteeism of more than 8 hrs per menstrual cycle (23), impairing productivity. Moreover, indirect costs of absenteeism and presentism were estimated over $4,000 usd annually when premenstrual symptoms are presented (24). The scenario might get worse considering that a menstruating person may experience around 480 menstrual cycles during their reproductive life, and the potential negative impact of PMDD becomes even more significant. In line with this idea, women with PMDD reported a general decline in health, poorer sleep quality, increased alcohol consumption, heightened anxiety and depression, a disrupted work-life balance, lower levels of psychological resilience, and increased perceived work demands (25). The overview of PMDD burden is given mainly by studies conducted in developed countries. While these studies have revealed significant data demonstrating the actual burden of PMDD, this disorder has been overlooked in developing countries within Latin American regions. Moreover, to this day no clinical attention or treatment is well known to be effective for this condition to dampen the negative impact of PMDD. Treatment options for PMDD may include ovarian suppression with gonadotropin-releasing hormone or oophorectomy but typically include pharmacotherapy with selective serotonin reuptake inhibitors (SSRIs) (26), dietary and nutritional interventions (27), as well as lifestyle changes and psychotherapy (28). Access to these treatments may vary depending on factors such as healthcare infrastructure and socioeconomic status within different Latin American countries.

## 4 The burden of cultural and gender perspectives

PMDD is a complex entity (29): from its not fully understood etiology to the influence of cultural perspectives. While menstruation is a natural biological process that indicates healthy coordination between the brain, the ovaries, and the uterus (30), the narrative of menstruation is surrounded by cultural perceptions, that may stigmatize it as taboo or shameful (31, 32). A recent review exhaustively explored societal, cultural and religious beliefs that contribute to taboos in sexual health, leading to secrecy, isolation, myths and misconceptions (33). These can result in inadequate menstrual hygiene management, missed educational opportunities, and perpetuate gender inequality. The social context can influence how individuals experience and express premenstrual symptoms. In rural areas, menstruation was often treated as a private or taboo topic when compared to urban areas, which discouraged open discussions and healthcare consultations when needed (34).

It is known that negative attitudes towards menstruation, often rooted in sexism, can lead to feelings of rejection and embarrassment (35). Following Marván et al. (35) perceptions and attitudes towards menses predict affective symptoms. Having a psychiatric diagnosis comes with a social stigma that negatively impacts self-concept and impairs recovery (36). Now, if we talk about a psychiatric disorder related to menstruation, the stigma around might be overwhelming. Due to double stigma (from mental health disorders and menstruation), the prevalence of PMDD is likely underestimated, particularly in countries with more traditional views and practices on gender, such as those in Latin American regions.

Gender perspectives influence even professional clinicians (37, 38). Regarding PMDD, a previous study found that female gynecologists are more frequently engaged in treating premenstrual mood disorders compared to male practitioners, and females tend to use prospective diagnoses in a higher proportion than men (39). Authors suggest that female gynecologists tend to stick to diagnosis guidelines for PMDD more rigorously than males, perhaps due to a more professional optic of the clinical condition. Healthcare professionals need to be aware and sensitized to detect premenstrual-related symptoms and to warrant accurate diagnosis. Otherwise, misdiagnosis or subdiagnosis could lead to invalidation of the negative experience and add more stress to a situation lacking empathy and denying access to treatments (as there would not be any condition to treat).

## 5 The burden of lack of sex-gender perspective in the healthcare system

Women’s mental health has been marginalized and dismissed as exaggerated and/or insignificant throughout history (11). For years, preclinical and clinical health research has primarily focused on male subjects across different species (40), often excluding females from scientific findings for several reasons, including concerns about hormonal fluctuations that may *interfere* with the results. This exclusion has led to a significant gap in our understanding of sex-specific health differences. Experimental designs have also frequently omitted biological sex as a co-variating factor, failing to consider potential biological variations. For example, steroid hormonal fluctuations such as estradiol and progesterone occur each 28-32 days in human females (41) while in males, androgens fluctuations occur in 24 hours (42), and these hormones impact on brain functions in both sexes. Then, it is crucial to understand the biological substrates underlying some pathologies due to sex differences impact on the prevalence, clinical manifestations and progression of the diseases (43). Consequently, diagnostic and treatment guidelines are mainly based on research conducted primarily in male subjects.

Female-specific conditions are beyond reproductive health. Historically, women’s health has been restricted to gynecological and obstetric issues, and other than these, are either invisible or not important enough (44). In the USA, the policy of including women in clinical research trials was stated in 1993, however, it was not until 2014 that the inclusion of sex as a biological variable (SABV) was mandatory in research funded by NIH (40). Despite the advancements in policies, there is still a long way to go, particularly in mental health research. A previous report that surveyed papers on psychiatric research revealed that only 19% of studies during 2009-2019 included an adequate design to elucidate potential sex differences (45). Worryingly, reports studying only females are barely 5% (46), suggesting that female-specific pathologies are scarcely considered in health literature and are far from being fully understood. Omissions of females in health-related scientific literature have stressed the disparities and bias in the healthcare system, often experiencing misdiagnosis or underestimation of symptoms leading to undertreatment of health conditions. Further, Silverio (11) argued that treatments for women’s mental health should be based on evidence derived from studies focused specifically on women, rather than relying on studies using men as a model. Also, the author proposes the concept of *Female Psychology* that frames mental health positively by considering both periods of strength and distress within women’s life course (47). Although sex and gender are different constructs, sex can influence the societal conformation of gender and the characteristics typically assigned to men and women (40). Gender refers to social constructs and norms that influence roles, relationships, and power positions for all people across their lifetimes. All these variables are suitable for change, which is why it is proposed that gender should not be a binary term (43). Transgender people have been excluded and underrepresented in scientific literature and clinical research. To this date, there is no systematic data on the prevalence of transgender people and premenstrual mood disorders. The transgender community is often susceptible to attacks, discrimination, abuse, physical and psychological violence (48). Transgender people are also at economic disadvantage (49). Only 1 out of 10 transgender persons have formal employment leading to inequities and difficulties in health access (50). Therefore, we stress the need that intersectionality must be involved in mental health matters.

It is acknowledged that gender inequalities in the healthcare system come from fundamental disparities between men and women (51). Thus, for years it has been thought that particular female diseases, such as premenstrual disorders and postpartum depression are *normal and inherent* in women’s lives. It is time to stop the normalization of adverse experiences by incorporating a gender perspective into the healthcare system. Health professionals could make a difference in how women transit throughout their reproductive and non-reproductive years (11).

## 6 The burden of non-intersectional approaches

Approaches that do not consider intersectionality significantly affect the detection, prevalence, prevention, and treatment of Premenstrual Dysphoric Disorder (PMDD) because they overlook the multifaceted and interacting social identities that shape women’s experiences. Intersectionality (52) recognizes the overlapping systems of oppression, such as those based on race, gender, class, and sexuality. Based on the intersectionality concept, Purdie-Vaughns and Eibach (53) propose an “interactive model” that assumes “multiple subordinate identities”. Therefore, individuals with multiple marginalized identities experience heightened prejudice and discrimination. This may predispose to health problems, as a previous report found the highest prevalences of mental disorders in black women, while the lowest prevalence was found in white men (54). This suggests that gender and race may be contributing to mental health outcomes. Moreover, Smolen et al. (54) report that women were underemployed or underpaid respect to men independently of race. Thus, Julia Monárrez (55) urged the consideration of the interaction with other factors of social exclusion, such as racial, gender, and sexual discrimination, that exacerbate the disadvantages of poor and working-class women.

Then, by not adopting an intersectional approach, research continues to perpetuate a gap in understanding how multiple subordinate identities affect women’s mental health. As Pilver et al. (56) emphasized, the Office for Women’s Health Research has acknowledged the need for increase research on PMDD among ethnic minority women. Policies developed without an intersectional framework might fail to address the root causes of health inequities.

Non-intersectional approaches often produce generalized data that fail to accurately represent the specific subgroups of women who are most affected by PMDD. For instance, ethnic minority women are often underrepresented in studies (56), and their unique symptoms experiences are not adequately explored when included. Also, they show that subtle forms of discrimination (e.g., rudeness, unfair treatment) are significantly associated with PMDD in ethnic minorities (56). Without an intersectional optic, these nuanced experiences of minority women and their contributions to PMDD prevalence are overlooked, resulting in an incomplete understanding of the disorder’s epidemiology.

Prevention programs that do not account for the interplay of race, class, and other social determinants may fail to address the specific needs of subgroups. Several factors such as low income, malnutrition, and domestic violence are particularly relevant in developing countries (57). Intersectional approaches would tailor preventive measures to address these socioeconomic variables. Moreover, non-intersectional approaches neglect how stress and adversity are disproportionately experienced by rural or low-income populations, contributing to the dynamic nature of PMDD prevalence (58). Intersectionality would guide more targeted prevention strategies, focusing on mitigating stressors specific to these communities.

Regarding treatment, approaches neglecting intersectionality can perpetuate biases within the healthcare system. For example, low-income, underinsured, and minority women often encounter limited access to mental health resources and face higher discrimination in healthcare settings (59). This might lead to inadequate or inappropriate treatment due to a lack of understanding of their unique circumstances.

Adopting an intersectional perspective in treatment plans would consider the simultaneous impact of multiple forms of oppression and identity factors, leading to more comprehensive and personalized therapeutic approaches. Ignoring intersectionality can result in one-size-fits-all treatment paradigms that ignore the compounded adversities that disadvantaged groups face.

## 7 The burden of a vicious cycle

**Figure 1:**
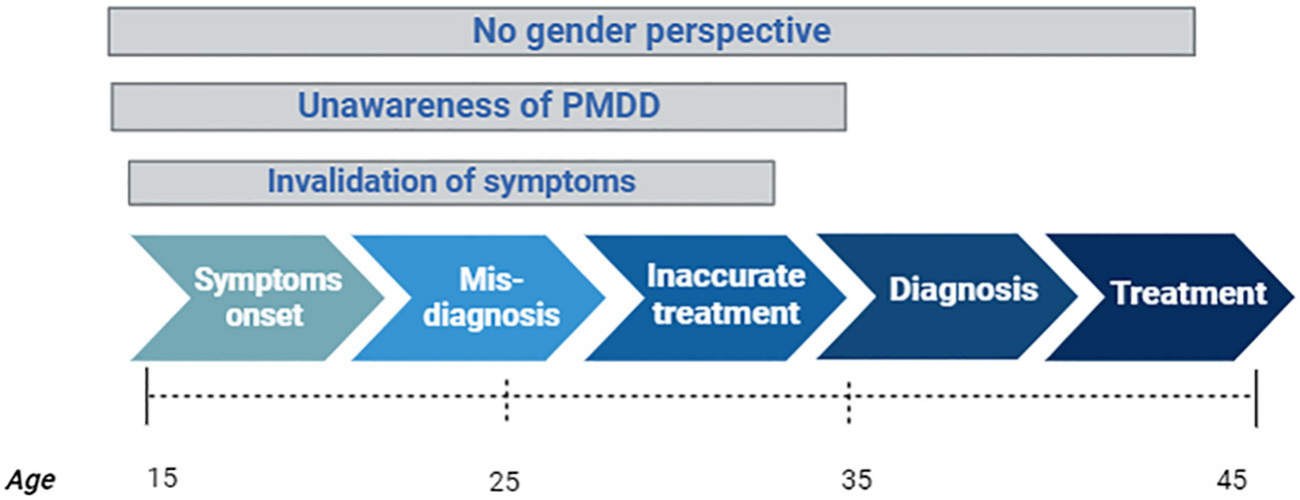
Pathway of individuals with PMDD symptoms and the impact of invalidation, unawareness and gender bias in each stage over time. Created with Biorender.com.

The overlook of premenstrual mood disorders contributes to the lack of data regarding the burden of illness. This may conform a vicious cycle: the absence of gender perspective leads to a lack of recognition which in turn leads to underestimation of the disorder’s prevalence. Lower prevalence results in reduced awareness among public authorities and decision-makers, leading to a lack of resources allocated for research on the epidemiology, etiology and treatment of the disorder. Without research, there’s no knowledge, and without knowledge, there’s no awareness. Worryingly, this vicious cycle is affecting women’s lives for more than 30 years, as symptoms usually begin around age 15, and misdiagnosis and mistreatment can last for 20 years before receiving the correct PMDD diagnosis. Even after diagnosis, the lack of gender perspective may lead to mistreatment, as PMDD symptoms may still be mistakenly attributed to the natural process of menstruation (10) (**Figure 1**).

The situation in Latin America is concerning. There have been no recent studies on the prevalence or burden of premenstrual disorders (PMDD) in the past decade. We still rely on outdated data from developed countries, which suggests that 8% of females of reproductive age suffer from PMDD (60). However, it is reported that adverse situations in the form of minimal health facilities, security not guaranteed, and high unemployment rates, are higher in developing countries with respect to developed countries (61). These adverse conditions can increase the risk of PMDD and worsen premenstrual symptoms (62). It is plausible then, that developing countries may have high rates of premenstrual mood disorders, beyond the 8% that we have historically considered.

## 8 Conclusion

Here, we have discussed the burden of aspects that worsen the experience of having PMDD in a non-gendered perspective health system that builds a barrier to understanding and properly addressing PMDD. It is undoubtedly the omissions and debts that people with PMDD face when they transit through this condition. From the invalidation of the symptoms to the ~20 years it takes to have a proper diagnosis. The delay in diagnosis can lead to prolonged psychological distress for those affected. The evidence (and the lack of it) highlights the crucial need to address gender perspective actions to bridge the inequalities within health systems that lead to improved mental health outcomes.

Here is a call to foster actions that overcome invalidation, minimization, underdiagnoses, misdiagnosis, and social stigma that surround premenstrual mood disorders. We can start by opening the conversation around menstruation and how unique this may be for every menstruating person.

We strongly appeal to implement actions that raise awareness and educate on PMDD, from healthcare providers, policy makers and the general population. Actions targeting public awareness through campaigns in public spaces, social media, among others, can help to destigmatize PMDD and encourage people to look for help if they are experiencing symptoms. Promote healthcare professionals to look at the unique experiences and particular needs of persons experiencing premenstrual symptoms. Health providers must receive proper training in gender-based care to positively impact in women’s lives. It is essential to encourage and fund further research into the neurobiological, psychological, and social factors that contribute to PMDD. We have to create useful knowledge to develop more effective diagnostic tools, preventive strategies and treatment options. The UK research agenda for PMDD (63) exemplifies a collaborative effort to identify key research priorities. Developed by a diverse group including individuals with PMDD, their families, healthcare professionals, researchers, support organizations, and emergency responders, the agenda prioritizes five key areas: diagnosis and management of PMDD, best approaches for psychological support, suicide and self-harm prevention, the impact of PMDD on life, and hormonal triggers for PMDD. Additionally, the agenda highlights several other potential research areas, such as the causes and biology of PMDD, destructive behavior, surgery and post-surgery support, barriers to support, the burden of PMDD, premenstrual exacerbation of existing disorders, neurodivergence, support from the welfare system, and PMDD education and training. This comprehensive approach underscores the multifaceted nature of PMDD and the need for multidisciplinary research to address its various aspects.

Finally, approaches that ignore gender perspective and intersectionality result in a fragmented understanding of PMDD, leading to ineffective prevention and treatment strategies that do not meet the nuanced needs of diverse groups of women. Recognizing and integrating intersectional factors are essential for creating comprehensive and equitable solutions in healthcare.
